# Value of Endoanal Ultrasound in the Comprehensive Management of Crohn's Disease-Associated Anorectal Fistulas: A Case Report

**DOI:** 10.7759/cureus.99861

**Published:** 2025-12-22

**Authors:** Alfredo S Abarca Magallon, Hector Norman Solares Sanchez, Gustavo Galicia Negrete, Oscar Coyoli Garcia, Agustín Castro Segovia

**Affiliations:** 1 Department of Coloproctology, Hospital Regional Licenciado Adolfo López Mateos, Instituto de Seguridad y Servicios Sociales de los Trabajadores del Estado, Universidad Nacional Autónoma de México, Mexico City, MEX; 2 Department of Colorectal Surgery, Hospital Regional Licenciado Adolfo López Mateos, Instituto de Seguridad y Servicios Sociales de los Trabajadores del Estado, Universidad Nacional Autónoma de México, Mexico City, MEX; 3 Department of Surgery, Hospital Regional Licenciado Adolfo López Mateos, Instituto de Seguridad y Servicios Sociales de los Trabajadores del Estado, Universidad Nacional Autónoma de México, Mexico City, MEX

**Keywords:** crohn’s disease (cd), endoanal ultrasonography, magnetic resonance (mr), sphincteroplasty, transsphincteric fistula

## Abstract

Crohn’s disease frequently involves the perianal region, where complex anal fistulas represent a challenging manifestation due to high recurrence rates and the risk of sphincter injury, as well as fecal incontinence. We report the case of a woman with long-standing Crohn’s disease, who developed a complex anal fistula in which endoanal ultrasound (EAUS) was essential for accurate anatomical characterization, assessment of sphincter involvement, and therapeutic planning. Following medical optimization and sphincter-sparing surgery, the patient achieved complete healing with preserved continence.

This case highlights the value of EAUS as an effective, accessible, and cost-efficient imaging modality for the evaluation and follow-up of perianal Crohn’s disease, supporting individualized management strategies in selected patients.

## Introduction

Crohn’s disease is a chronic inflammatory bowel disorder that can affect any segment of the gastrointestinal tract, from the mouth to the anus. Extra-perianal manifestations - including ileocolonic inflammation, strictures, and enterocutaneous fistulas - often coexist and influence overall disease management, with complex anal fistulas constituting one of the most difficult complications to treat because of their tendency to recur and their potential impact on continence [1-3]. Precise anatomical evaluation and timely, coordinated management are essential to achieve long-term healing while preserving sphincter function [1-3].

Imaging has become fundamental for treatment planning. Colonoscopy defines luminal activity and associated lesions, whereas perianal mapping allows identification of the primary tract, internal opening, secondary extensions, and their relationship to the sphincter complex [4-6]. Biologic therapy has transformed the natural history of fistulizing disease by inducing and maintaining closure of fistulas, thus allowing surgery to be performed under more favorable conditions [7,8]. Sphincter-sparing procedures are prioritized according to anatomy, rectal inflammation, and functional status as assessed with validated continence scores [9,10].

Endoanal ultrasound (EAUS) provides high-resolution, real-time visualization of the internal and external sphincters and the puborectalis muscle, enabling identification of the internal opening, fibrosis, and postoperative changes. It complements pelvic magnetic resonance imaging (MRI), which remains the reference standard for complex cases [11,12]. The Parks classification continues to guide decision-making by describing the anatomic course of fistulas and estimating the risk of continence [13]. Cross-sectional imaging recommendations from recent guidelines consolidate MRI as the standard for defining activity and extension, while maintaining complementarity with EAUS in daily practice [14]. Narrative reviews synthesize an optimal algorithm that includes control of sepsis, medical optimization, and individualized, sphincter-preserving closure, while reserving advanced or emerging options for refractory disease [15,16].

Within this framework, contemporary evidence supports EAUS as an effective, accessible, and cost-efficient modality for detailed sphincter and tract assessment, for postoperative monitoring, and for follow-up. In selected cases, it achieves diagnostic performance comparable to MRI, reinforcing its value in the comprehensive management of Crohn’s disease-associated anorectal fistulas [17-19].

Despite the widespread use of MRI as the reference standard for perianal Crohn’s disease, the specific contribution of EAUS to therapeutic decision-making, functional preservation, and postoperative assessment remains underreported. This case is presented to illustrate how EAUS can complement established imaging modalities by providing detailed sphincter evaluation and supporting individualized, sphincter-sparing management in selected patients.

## Case presentation

A 63-year-old woman with a long-standing history of Crohn’s disease, treated with mesalamine and infliximab, presented with anal pain, intermittent purulent discharge, and occasional minor bleeding. Physical examination revealed a posterior secondary opening, approximately 2 cm in diameter, located 3 cm from the anal verge, and draining seropurulent material. A palpable internal opening was detected at the posterior midline. Digital rectal examination showed normal tone and caliber, without palpable collections.

A biopsy specimen obtained during colonoscopy confirmed chronic inflammation with lymphoplasmacytic infiltrate consistent with Crohn’s disease.

**Figure 1:**
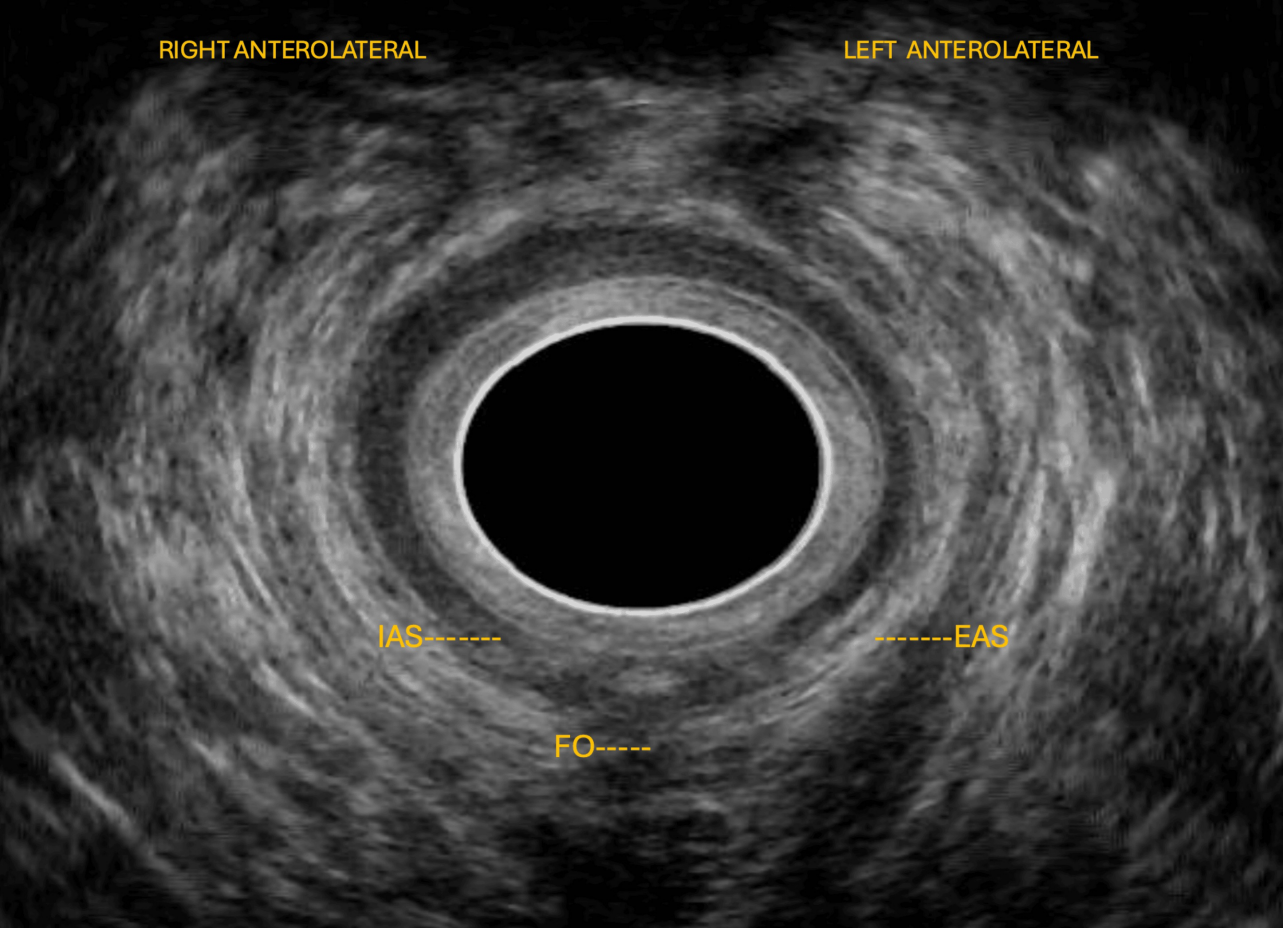
High posterior transsphincteric fistula on 360° endoanal ultrasound Endoanal ultrasonography demonstrates a high posterior transsphincteric fistulous tract, measuring approximately 18 mm in length, and extending toward the puborectalis muscle. The tract traverses the external anal sphincter, with partial involvement of both anterior and posterior quadrants. Areas of fibrosis and partial internal sphincter disruption are noted, without evidence of abscess or fluid collection. The labeled endoanal ultrasound (EAUS) image shows the internal anal sphincter (IAS), the external anal sphincter (EAS), and the fistulous opening (FO).

EAUS revealed a high posterior transsphincteric fistula, approximately 18 mm in length, extending to the puborectalis muscle, with partial involvement of the external anal sphincter in the anterior and posterior quadrants, along with areas of fibrosis and partial disorganization of the internal sphincter, and without evidence of abscess or collections (Figure 1).

These findings established the diagnosis of a transsphincteric anorectal fistula associated with Crohn’s disease, in a controlled inflammatory phase.

Initial management included medical optimization with antibiotics (ciprofloxacin and metronidazole), anti-inflammatory therapy with mesalamine, and biologic treatment using infliximab, which was continued throughout the perioperative period [20].

**Figure 2:**
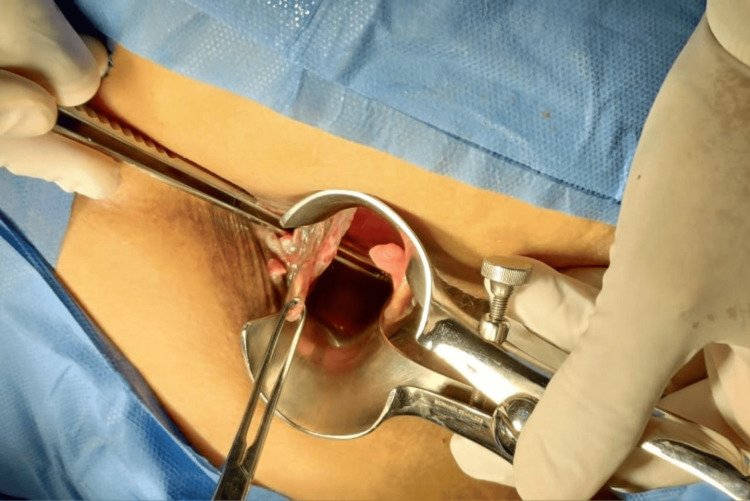
Intraoperative confirmation of the fistulous tract Introduction of a metallic stylet through the external opening confirmed the high posterior right transsphincteric tract, previously identified by endoanal ultrasound. The stylet delineates the trajectory toward the internal opening, located at the posterior midline, facilitating accurate identification and complete excision of the tract.

**Figure 3:**
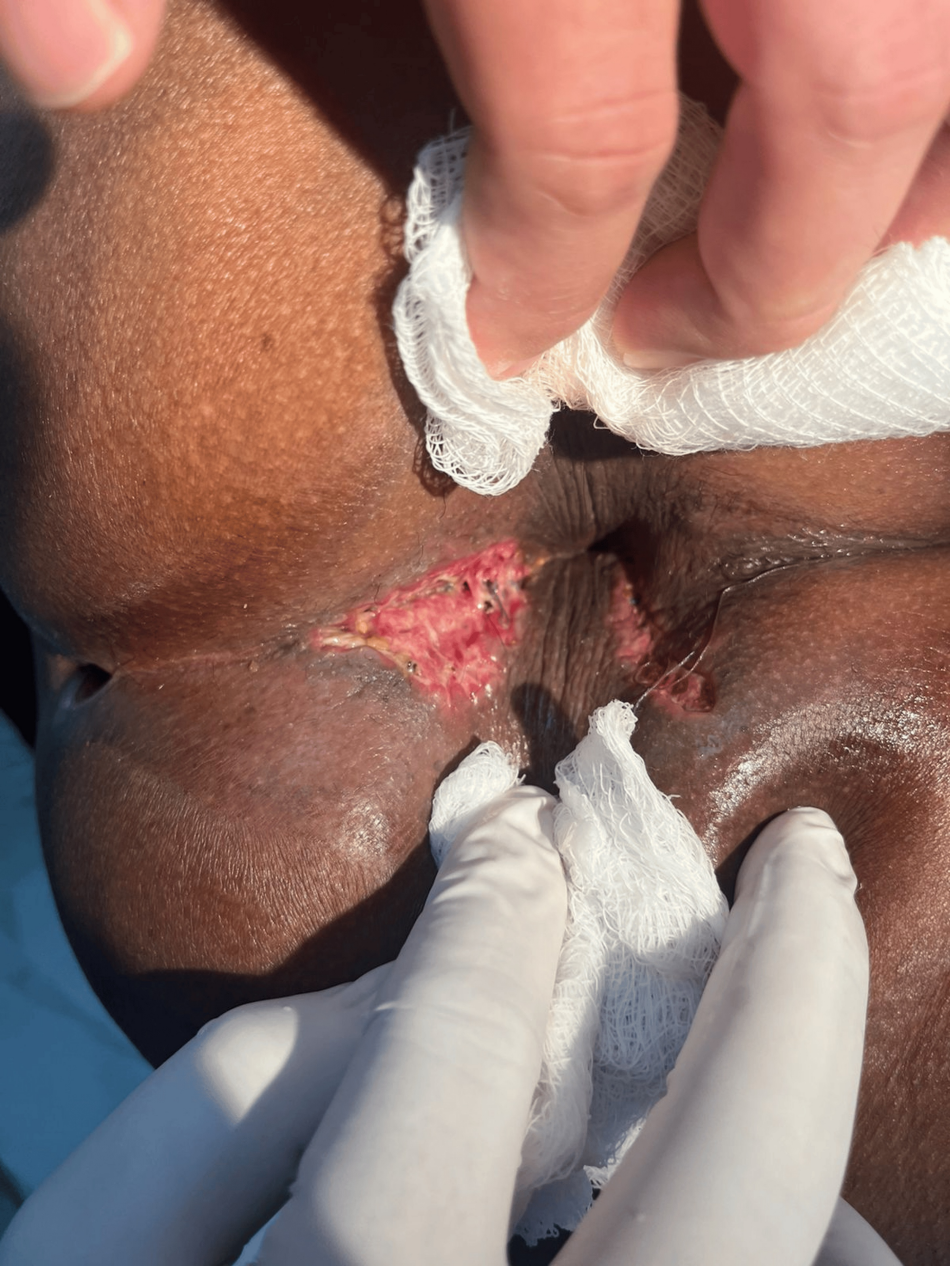
Early postoperative appearance Early postoperative appearance of the surgical site showing healthy granulation tissue and adequate secondary healing, without evidence of infection or complications.

After medical optimization with anti-TNF therapy and confirmation of quiescent mucosal disease, elective surgical management was undertaken. The procedure began with the introduction of a metallic stylet through the external opening, confirming the course of the posterior right transsphincteric tract, previously demonstrated by EAUS (Figure 2). The tract was subsequently excised, and the wound was left open for drainage and secondary healing. During early postoperative follow-up, the surgical site showed adequate granulation tissue, without signs of infection (Figure 3).

**Figure 4:**
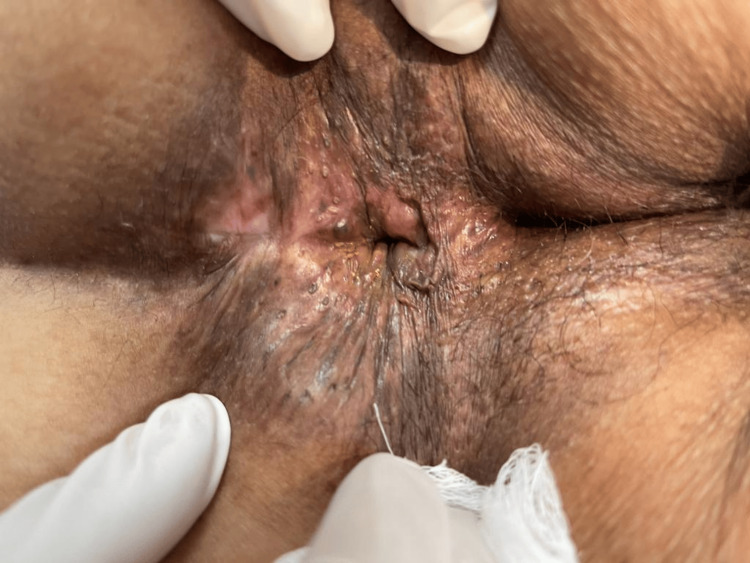
Follow-up evaluation Follow-up evaluation demonstrated complete epithelialization and satisfactory healing of the surgical site, with no recurrence or continence impairment.

The postoperative course was uneventful. The patient reported complete resolution of pain and discharge and normalization of bowel habits to one to two formed stools per day (Bristol Type 4-5), without bleeding or tenesmus. On subsequent follow-up, complete epithelialization of the surgical bed was observed, with a normotonic anal canal and no residual openings (Figure 4). The Jorge-Wexner continence score improved from 12 preoperatively to 2 postoperatively, confirming preserved continence and sustained functional recovery [10].

## Discussion

The management of perianal fistulas associated with Crohn’s disease requires a multidisciplinary and sequential approach. Current guidelines emphasize the need to achieve systemic inflammatory control and sepsis drainage before attempting definitive closure, integrating medical and surgical strategies in a coordinated fashion [1-3].

Accurate diagnostic stratification is essential for selecting an appropriate sphincter-preserving technique. Endoscopic evaluation defines luminal activity and concomitant lesions [4], while imaging provides the anatomical mapping required for planning. The ESGAR (European Society of Gastrointestinal and Abdominal Radiology) consensus highlights the complementary role of EAUS and pelvic MRI in assessing fistula-in-ano and other sources of anal sepsis [5]. Likewise, the global perianal Crohn’s disease consensus recommends reserving definitive surgical repair for patients with controlled rectal inflammation, after complete anatomical assessment [6].

Biologic therapy forms the foundation of treatment. Pivotal trials demonstrated that infliximab promotes fistula closure and maintains long-term response, changing the prognosis of fistulizing disease [7,8]. This medical optimization allows surgery under improved biological conditions, reducing recurrence risk.

Among surgical options, the preservation of continence remains the priority in complex fistulas. Sphincter-sparing techniques, such as endorectal advancement flap and ligation of the intersphincteric tract (LIFT), achieve comparable healing rates, with a lower potential impact on continence in favor of LIFT [9]. Functional assessment, with validated scoring systems including the Jorge-Wexner scale, remains a useful tool to evaluate baseline function and postoperative evolution [10].

EAUS provides a detailed visualization of the sphincter complex and plays a central role in the follow-up of perianal Crohn’s disease. It allows real-time assessment of the fistulous tract, healing progress, and early detection of subclinical recurrence in a cost-effective manner [11]. Combined medical and surgical management yields higher remission rates and lower recurrence compared with either strategy alone [12]. Anatomical classification systems, such as that proposed by Parks, continue to guide surgical decision-making by defining the extent and type of fistula, and its relationship with the sphincter apparatus [13].

Cross-sectional imaging guidelines reaffirm MRI as the standard for evaluating activity and extension, yet emphasize its complementarity with EAUS in daily clinical practice [14]. Contemporary reviews summarize the optimal therapeutic sequence as sepsis control, biologic optimization, and conservative closure of the individualized tract, reserving advanced approaches (e.g., laser therapy, glues, and cellular treatments) for refractory or recurrent cases [15,16].

In comparative literature, EAUS demonstrates diagnostic performance similar to MRI for delineating fistulous anatomy and identifying internal openings, with additional advantages of lower cost, accessibility, and suitability for serial monitoring. Buchanan et al. reported that, although MRI remains superior for mapping complex extensions, high-frequency EAUS shows substantial anatomical concordance and can replace MRI in less extensive cases [17]. Siddiqui et al. found comparable global sensitivity between both modalities, supporting EAUS as a first-line diagnostic study when anatomy is accessible to the probe [18]. More recent meta-analyses using three-dimensional EAUS confirm high overall accuracy, consolidating its current role in routine practice [19].

The present case illustrates the effectiveness of this sequential algorithm: inflammatory control, precise imaging-based mapping, and tailored sphincter-preserving repair, leading to sustained healing and functional preservation.

## Conclusions

This case demonstrates that successful management of Crohn’s disease-associated transsphincteric fistulas relies on detailed anatomical assessment, control of systemic inflammation, and timely sphincter-preserving surgery. EAUS proved to be a valuable adjunct by providing precise evaluation of sphincter involvement and fistulous anatomy, supporting individualized surgical planning and preservation of continence.
